# Low prevalence of end plate junction failure in danish patients with lumbar disc herniation

**DOI:** 10.1038/s41598-020-74690-w

**Published:** 2020-10-19

**Authors:** Søren Francis Dyhrberg O’Neill, Jonas Morten Fidelman, Linne Steinar Haarup, Christian Lund, Mikkel Brunsgaard Konner

**Affiliations:** 1 Spinecenter of Southern Denmark, Lillebælt Hospital, Middelfart, DK5750 Denmark; 2 University of Southern Denmark, Institute of Regional Health Science Research, Odense, DK5230 Denmark; 3 Department of Sport Science and Clinical Biomechanics, University of Southern Denmark, Odense, DK5230 Denmark; 4 Department of Radiology, University Hospital of Southern Denmark, Vejle, DK7100 Denmark; 5 Spinecenter Djursland, Grenå, DK8500 Denmark

**Keywords:** Musculoskeletal system, Computed tomography, Magnetic resonance imaging

## Abstract

The present study was undertaken to determine the prevalence of endplate junction failure in a smaller cohort of Danish patients with lumbar disk herniation and compare this to the previously published data from India. Consecutive patients seen in a large regional hospital spine-care unit, with a clinical presentation suggesting a lumbar disk herniation with concomitant radiculopathy and confirmatory recent MRI were included. Additional imaging by CT was performed as part of the study and these were analyzed with specific attention to endplate junction failures. For ethical reasons, the number of participants was kept to a minimum and a total of 26 patients were included. The prevalence (n = 5) of endplate junction failure was found to be statistically significantly lower than that previously reported. Our findings do not echo those previously reported in an Indian population: Endplate junction failure was indeed observed, but at a significantly lower rate. We discuss potential reasons for the difference in findings with due attention to the weaknesses of the current study.

## Introduction

When dealing with low-back pain, more often than not, the etiology and underlying pain mechanisms are unknown or at best speculative. Such spinal pain conditions are often labeled as ‘non-specific’, ‘biomechanical’ or ‘degenerative’ back pain, despite the questionable significance of degenerative changes and biomechanical function in individual cases. A notable and relatively common exception is the intervertebral disk (IVD) herniation with concomitant radiculopathy, which has characteristic symptoms and a well-understood pathology. At least, that was the situation in 2013 when Rajasekaran et al.^1^ reported that lumbar IVD herniation was more commonly due to endplate-junction failure (EPJF) than rupture of the annulus fibrosus (AF). The paper was a notable challenge of the established wisdom and it deservedly won the 2013 ISSLS prize (International Society for the Study of the Lumbar Spine).

The common understanding of IVD herniation as a structural defect in the AF permitting herniation of the nucleus pulposus (NP) into the spinal canal, can be traced back to the seminal paper by Mixter and Barr^2^. Mixter and Barr noted that “There is often a history of trauma not immediately related to the present condition”, but in a case series by Mixter and Ayer^3^ the following year, they reported that “In only [..] one-third of all the cases did it seem certain that an accident was followed immediately by the symptoms for which later the operation was performed.” It thus seemed clear from early on, that the etiology of IVD herniation was more complicated than acute traumatic rupture of the AF.

The current understanding of the pathophysiology of IVD herniation is more detailed and degeneration of the IVD is understood to be a prerequisite for the development of IVD herniation in most cases (see Inoue and Espinoza for a review^4^). Traumatic rupture of the AF in an otherwise healthy and non-degenerated disk is a rare occurrence, and even in children and adolescents where disk herniation is very rare but typically associated with trauma, it seems the trauma typically acts as a trigger of herniation in an already predisposed disk^5^. In a previous study in the United States of 31 patients below the age of 21, who underwent surgery for lumbar disk herniation, Banerian et al.^6^ found 6 cases (19%) of endplate avulsion fractures.

When a rupture of the AF presents radiologically as a disk herniation, it has most likely been preceded by progressive disk degeneration, initiated by age related changes and/or some other traume. E.g. it seems, that disruption of the vertebral endplate has greater impact on the mechanical properties of the disk and the development of degeneration, than injury to the AF^7^. Conversely, once disk degeneration is established it has deleterious effects on the mechanical properties of the AF^8^ which may lead to AF disruption. In other words, the chain of events which lead to IVD herniation is likely to be initiated by either injury to the vertebral endplates^9^ or age related disk degeneration, which in turn leads to altered distribution of mechanical forces within the disk, as well as weakening and rupture of the AF, resulting finally in disk herniation. In vitro studies of geriatric cadavaric motion segments^10^ suggest that that once disk degeneration with decreased intra-discal pressure has been established, tensile loads sufficient to cause structural failure may well affect the disk-bone junction predominantly, which resonates well with the findings of Rajasekaran et al.^1^.

The findings of Rajasekaran et al.^1^ thus do not challenge the chain of events leading to IVD herniation as such, but rather the frequency of the anatomical structures involved once the herniation manifests. The radiological findings on which those conclusions were founded, were vertebral body defects such as avulsion fractures of the cartilaginous rim with/without subchondral bone. Presumably, such changes with bony defects would be more readily visible on plain X-ray and Computer Tomography (CT) imaging with its relatively high contrast, but the standard imaging procedure on clinical suspicion of IVD herniation is Magnetic Resonance Imaging (MRI). MRI has relatively poor contrast and resolution compared to both plain X-ray and CT, which arguably could explain why such avulsion fractures might be overlooked and rarely described in radiological report of MRI findings.

The clinical management of lumbar disk herniation is guided by the natural history of the condition and the response to conservative treatment whilst monitoring for the development of significant neurological deficits^11^. To our knowledge, there is presently no rationale for stratifying patients into different management strategies on the basis of the classification of herniation morphology as described by Rajasekaran et al.^1^. None the less, the morphological classification of herniation’s may prove clinically relevant in future and thus the prevalence of the different subgroups is worth investigating.

The aim of the present study was estimate the prevalence of EPJF in a consecutive cohort of Danish patients with lumbar disk herniation and radiculopathy, and compare it to that reported by Rajasekaran et al.^1^. The null-hypothesis was that the prevalence of EPJF in Danish patients was similar to that reported from India^1^.

## Methods

Consecutive patients were recruited from the Spinecenter of Southern Denmark, a large regional hospital department (Lillebaelt Hospital, Middelfart) serving a population of approximately 1.2 million. The department has both a surgical and medical section and the patient population is constituted mainly of individuals with persistent spinal pain syndromes which have failed to respond to relevant treatment in the primary care sector. Referrals are accepted from general medical practitioners, chiropractors, and medical consultants, as well as other hospital departments in the region.

Patients were included in the present study on the basis of a clinical diagnosis of lumbar radiculopathy and an MRI confirmed disk herniation at the corresponding side and segmental level (see details below).

General inclusions criteria:

1. Age between 20 and 60 years.
2. Body Mass Index <30.0.
3. Fluent in written and spoken Danish.
4. Single segment disk herniation on MRI.
5. Side and segment of disk herniation (MRI) in concordance with clinical examination findings.
6. MRI performed and read/reported at Vejle or Middelfart hospitals (in accordance with department guidelines).
7. Date of MRI no more than 3 months before the study CT scan.

General exclusion criteria

1. Competing spinal diagnoses such as central canal stenosis, tumors, gross vertebral fractures, etc.
2. Contraindication to CT scanning such as known or suspected pregnancy.
3. Previous surgery in the lumbar region.

### Inclusion

Patients who met the inclusion criteria were informed of the details of the study and invited to participate. Those who expressed an interest in taking part in the study, were sent the full participant information which included a description of the potential risks associated with ionizing radiation, as per the science ethics committee approval. Informed consent was secured from all participants before enrollment in the study.

### Evaluation of images

The MRI images were read and described as per the local hospital guideline, which adheres to the recommendations of the North American Spine Society and American Society of Spine Radiology^12^.

The CT scans were read and described by an experienced musculoskeletal radiologist-chiropractor (CL) with post-graduate radiological training and 9 years of full-time experience assessing spinal imaging in the radiology department of Lillebaelt Hospital, Vejle. The CT scans were categorized according to the nomenclature described in the Rajasekaran study, i.e. as Type 1 sub-types A–D (EPJF) or Type 2 (AF rupture).

### CT scanning protocol

The CT scans were performed as per protocol by the local radiology department (Lillebaelt Hospital, Vejle) on a General Electric VCT 64 slice scanner (kVolt: 120, mAmp: 600 + dosemod 20% ASIR, time: 3–4 s per segment, dependent on z-length, 0.6 s/rotation, collimation: 0.625 mm, coverage: 40 mm, slice thickness: 0.625 mm, post-processing slice thickness: 0.9 mm in all planes, pitch: 0.984:1, 0.6 s/rotation, coverage speed: 62.62 mm/s, scan series: 2 scout, 1–2 helical technique scans).

### Analysis of data

Results are presented as simple frequency counts (number of observations in each CT category). Fisher exact test ($5\times 2$ table) was used to compare the data from our cohort with the frequency counts of Rajasekaran et al.

Data was analyzed using R (v 3.4.4, platform x86_64-pc-linuc-gnu) (R Core Team (2018). R: A language and environment for statistical computing. R Foundation for Statistical Computing, Vienna, Austria. URL https://www.R-project.org/).

### Ethics and consent

The local science ethics committee for the Region of Southern Denmark approved the study (Project-ID: 20140106). Informed consent was obtained from all participants. The study was conducted in accordance with the Helsinki declaration.

## Results

A total of 33 patients were included, but 6 did not show up for the CT-scan and were excluded from further analysis. One case was originally described as a disk herniation on MRI, but was re-classified as a disk protrusion (with annular calcification), as per the nomenclature recommendations^12^, leaving 26 participants in the study.

Summary statistics of the participants are presented in Table 1. Summary statistics of the CT categories are presented in Table 2.

**Table 1:** Summary table of participants.

Sex	n	Age
Female	4	47 [13.5]
Male	22	49 [10.8]

Age listed as mean [sd].

**Table 2:** Summary table of CT findings.

	1a	1b	1c	1d	2
Current data	2	3	0	0	21
Rajasekaran data	30	46	24	4	64

Frequency (count) of CT categorization (1a–1d: Subtypes of endplate junction failure, 2: Rupture of the annulus fibrosus). Note: In one case of the current data, a calcific density in the spinal canal immediately posterior to the vertebral corner on CT was described as ‘most probably calcification of the AF’ rather than an actual EPJF. It has however, been categorized here as a type 1b avulsion.

When comparing the current data (frequency count) to those reported by Rajasekaran et al.^1^, Fisher exact test yielded a $P=0.002$.

**Figure 1:**
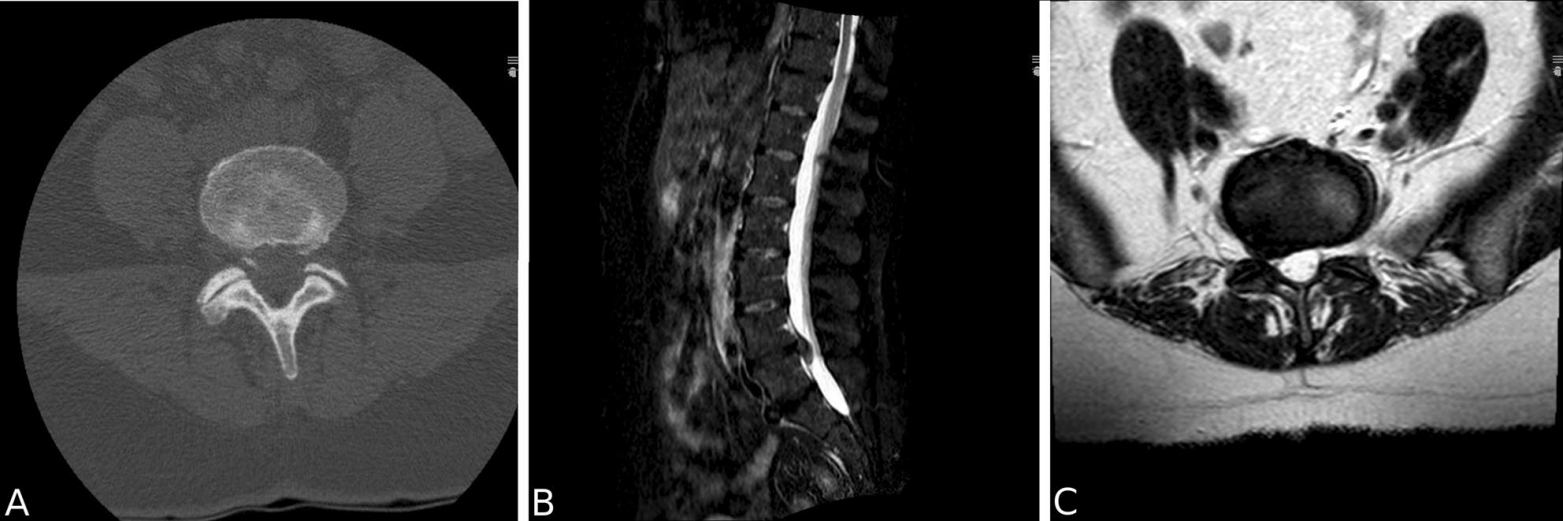
Sample CT and MR from one individual. (**A**) CT Axial with type 1b bone avulsion L4/L5. (**B**) STIR sagittal with disk extrusion L4/L5. (**C**) T2 axial with right disk extrusion L4/L5.

## Discussion

Our findings do not echo those of Rajasekaran et al.^1^. We found a relatively low prevalence of EPJF with only two of type 1a, three of type 1b (of which one was probably an AF calcification) and none of type 1c or 1d. Conversely, AF rupture (type 2) was by far the most prevalent in out data. For an illustration of a type 1b avulsion, see Fig. 1.

Despite the relatively small number of participants in the present study, formal statistical testing indicate that findings like ours are unlikely to arise by chance in the distribution reported by Rajasekaran et al. In other words, it is more likely that our patients are drawn from a different population distribution or, alternatively, that significant differences in the methods used to categorize patients affected our results. These two possibilities are discussed below.

The inclusion criteria described by Rajasekaran et al.^1^ were *consecutive patients requiring a single level discectomy*, but it is not entirely clear which clinical or radiological criteria formed the basis of the initial diagnosis of disk herniation and thus inclusion in the study. We suspect it was a judgment call by an experienced clinician and we can not rule out that different inclusion criteria have played a part in the observed difference in findings.

Other obvious differences exist between the study populations: The study by Rajasekaran et al was conducted in India, whereas the present study was conducted in Denmark. A review of the literature suggests that as much as 74% of the variation in adult lumbar disk degeneration is hereditary, whereas exposure to physical loading plays a lesser role^13^. Genetic population differences between India and Denmark could be important determinants of lumbar disk degeneration and thus the risk of IVD herniation and therefore also of the differences observed between the current study and that of Rajasekaran et al.

As mentioned in the introduction, tensile loads on an already degenerated disk may place the disk-bone junction under particular stress^10^ and therefor the mechanical characteristics of the bony tissue and the fibrous connections may be important determinants of the type of tissue damage seen in disk herniation. Vitamin D status may play an important role in this context, as the link between vitamin D levels and bone mineralization is well established (for a recent review see van Driel and van Leeuwen^14^). It has been reported that a North–South gradient can be observed in Europe, with Scandinavians having higher serum vitamin D levels and a mean vitamin D intake twice that of other European countries. Conversely, vitamin D deficiency was observed in more than 30% of the population in India, with higher prevalence in larger cities^15–17^. The variation in vitamin D status may be partly determined by genetics, nutrition, skin color, sun light exposure and other factors. Regardless of the underlying reasons, a difference in Vitamin D status and bone mineralization would conceivably affect the likelihood of bony and cartilagenous avulsion of the vertebral endplate junction under tensile loads and therefor explain the observed difference in prevalence of endplate junction failure (EPJF).

As mentioned, there may be differences in the inclusion criteria and procedures in this study and that of Rajasekaran et al which could make direct comparison more difficult. The participants in both studies however, were recruited from a cohort of patients where a clinical suspicion of disk herniation with radicular pain had prompted relevant imaging, which in turn confirmed the suspicion. In this regard, the two patient groups are comparable.

Another methodological consideration is that calcification of herniated disk material could be radiologically misclassified as avulsed bony fragments, and vice versa. Previous publications^18^ have reported that microscopic calcification in the excised nucleus is common in patients operated for lumbar disk herniation and calcification of the annulus fibrosus is also relatively common^19^. The study by Rajasekara et al included microscopic analysis of the excised tissues which further supported their conclusions of a high prevalence of avulsed bone and cartilage. We are not convinced, that the difference in findings can be explained satisfactorily by misclassification of avulsed fragments as calcification of herniated disk tissue and vice versa.

The present study included only a relatively small number of participants, which at first must be considered a weakness and potential risk for a type 2 error. However, the numbers were sufficient to reject the null-hypothesis at a *P* level of 0.002. Ethical considerations imposed a limit on the number of participants in the present study, not only because of the potential carcinogenic effects of ionizing radiation, but also because CT-verified EPJF in disk herniation patients has no immediate clinical consequences, beyond those of disk herniation identified on MRI.

The present study was designed to examine the prevalence of EPJF in Danish patients compared to the data previously published, but it does not allow for any scientific examination of the underlying reasons for the difference we observed. The preceding discussion of genetics, vitamin D status etc as potential explanations of the observed difference, is essentially speculative.

In a smaller consecutive cohort of Danish patients with clinical and MRI confirmed intervertebral disk herniation, endplate junction failure evident on CT was a relatively uncommon finding, and one which was significantly less prevalent than that reported in India.

The reasons for this disparity are not clear, but we would argue that it is too soon to discard the original description of disk herniation as an annular rupture and replace it with one of predominantly bony and cartilaginous avulsion. The anatomy of failure in lumbar disk herniation may prove to vary between clinical contexts.
